# Evaluation of primary health care system in Yangon Region, Myanmar: a mixed-method approach

**DOI:** 10.1093/eurpub/ckac131.016

**Published:** 2022-10-25

**Authors:** J Moon, SJ Kang, JW Noh

**Affiliations:** 1 Graduate School of Medical Sciences, University of Groningen, Groningen, Netherlands; 2 Institute of Health and Environment, Seoul National University, Seoul, South Korea; 3 Division of Health Administration, Yonsei University, Wonju, South Korea

## Abstract

**Background:**

Many low- and middle-income countries and international organizations have invested resources to strengthen primary health care services. Despite efforts from the Ministry of Health on primary health care, barriers to accessing health care services and health inequality in Myanmar still exist. This study aimed to identify the challenges and unmet needs in the current primary health care services by assessing the experiences and perceptions of healthcare workers and local leaders in three townships (Htantabin, Hmawbi, and Taikkyi) in Yangon, Myanmar.

**Methods:**

The study was conducted among healthcare professionals and community leaders in three townships. By adopting a mixed-method approach, a cross-sectional health needs assessment survey was conducted for quantitative data (n = 66), and focus group discussions (15 group discussions) were conducted online for qualitative data.

**Results:**

As a result of the survey regarding six domains; hygiene, primary medical care, maternal and child health, infectious diseases, non-communicable diseases, and leadership, enhancing the management and leadership capacity had the lowest average score on the current achievement (2.81 out of 5), while strengthening infectious disease control service and accessibility was perceived as the highest mean on the priority of intervention (4.28 out of 5) and the impact of the intervention (4.7). The focus group discussions revealed that while specific infrastructures and equipment necessary for the category were addressed, the need for financial support has been the recurrent theme throughout the discussions.

**Conclusions:**

Utilizing the World Health Organization’s six-building block framework, our findings suggest that a long-term targeted financial investment in the primary health care system is critical in Myanmar by increasing health care expenditure per capita. At the same time, related barriers and facilitators should be considered to optimize the effectiveness of prioritized interventions.

**Key messages:**

• Health care providers and local leaders perceived the management and leadership capacity as the lowest current achievement.

• A long-term targeted financial investment in the primary health care system is critical in Myanmar.

